# Disentangling the influence of environmental and anthropogenic factors on the distribution of endemic vascular plants in Sardinia

**DOI:** 10.1371/journal.pone.0182539

**Published:** 2017-08-02

**Authors:** Mauro Fois, Giuseppe Fenu, Eva Maria Cañadas, Gianluigi Bacchetta

**Affiliations:** 1 Centro Conservazione Biodiversità (CCB), Dipartimento di Scienze della Vita e dell’Ambiente, Università degli Studi di Cagliari, Cagliari, Italia; 2 Dipartimento di Biologia Ambientale, ‘Sapienza’ Università di Roma, Roma, Italia; 3 Departamento de Botánica, Facultad de Ciencias, Universidad de Granada, Granada, Spain; Università di Pisa, ITALY

## Abstract

Due to the impelling urgency of plant conservation and the increasing availability of high resolution spatially interpolated (e.g. climate variables) and categorical data (e.g. land cover and vegetation type), many recent studies have examined relationships among plant species distributions and a diversified set of explanatory factors; nevertheless, global and regional patterns of endemic plant richness remain in many cases unexplained. One such pattern is the 294 endemic vascular plant *taxa* recorded on a 1 km resolution grid on the environmentally heterogeneous island of Sardinia. Sixteen predictors, including topographic, geological, climatic and anthropogenic factors, were used to model local (number of *taxa* inside each 1 km grid cell) Endemic Vascular Plant Richness (EVPR). Generalized Linear Models were used to evaluate how each factor affected the distribution of local EVPR. Significant relationships with local EVPR and topographic, geological, climatic and anthropogenic factors were found. In particular, elevation explained the larger fraction of variation in endemic richness but other environmental factors (e.g. precipitation seasonality and slope) and human-related factors (e.g. the Human Influence Index (HII) and the proportion of anthropogenic land uses) were, respectively, positively and negatively correlated with local EVPR. Regional EVPR (number of endemic *taxa* inside each 100 m elevation interval) was also measured to compare local and regional EVPR patterns along the elevation gradient. In contrast to local, regional EVPR tended to decrease with altitude partly due to the decreasing area covered along altitude. The contrasting results between local and regional patterns suggest that local richness increases as a result of increased interspecific aggregation along altitude, whereas regional richness may depend on the interaction between area and altitude. This suggests that the shape and magnitude of the species-area relationship might vary with elevation. This work provides—for the first time in Sardinia—a comprehensive analysis of the influence of environmental factors on the pattern of EVPR in the entire territory, from sea level to the highest peaks. Elevation, as well as other environmental and human-related variables, were confirmed to be influencing factors. In addition, variations of EVPR patterns at regional-to-local spatial scales inspire next investigations on the possible interaction between elevation and area in explaining patterns of plant species richness.

## Introduction

The question of how plant diversity is distributed on Earth has long fascinated and inspired biogeographers and ecologists. Due to the urgency of plant conservation and an increase in the amount of high resolution data available, many studies have explored how plant species’ richness results from interactions among topography, geology, climate and anthropogenic factors [1–3]. Nevertheless, global and regional patterns of plant richness are still in many cases unresolved.

Since endemic plants are frequently threatened, they constitute a pivotal group for conservation [4, 5]. Among endemics, plant species that have narrow extent of occurrence and area of occupancy deserve a priority for conservation, since they are often classified as threatened, mainly due to their limited number of locations/populations, genetic diversity and ecological amplitude [5, 6]. Despite their conservation interest, the ecology and distribution of such endemic plants has not yet been explored thoroughly, and further research is needed, especially in-depth studies at very fine scales [6–8]. Most research on this issue to date has been carried out on islands [9–11], which have long been considered global centres of plant endemism richness [12]. In studies on larger islands (including continental islands), the area *per se* contributed to explain most of species’ richness variation since population sizes usually increase with an island’s area, and thus extinction risk decreases [13]. But when plant diversity was analysed at increasingly fine scales, further influential factors were found. Generally, elevation gradient and habitat diversity were the most important drivers of plant distribution [3, 14]. Stuessy et al. [15] found the proportion of endemic species evolved through adaptive radiation to be positively related to habitat diversity on islands, assuming that speciation through adaptive radiation is much faster than random drift. In addition, environmental filtering along an elevational gradient differentiates ecosystems, leading to an increase of habitat diversity and isolation with elevation [3, 15]. Consequently, an increased speciation rate resulting in a larger percentage of endemic species can be expected for higher elevations. Support for this elevation-driven ecological isolation hypothesis comes from other islands in the Mediterranean region (e.g. Crete and Corsica), where an increase of the percentage of endemic species with elevation has been observed [3, 11]. Also, human beings are now considered one of the most novel forces in the evolution of life, since they are alarmingly increasing in the last decades, especially in lowlands [12, 16].

The five Mediterranean climate regions have been the site of many studies about endemic plant richness [3, 7, 9–11]. In the case of the Mediterranean Basin, where this study is focused, the diversification of several endemic plants across the islands of the Basin substantially originated via processes of land migration/vicariance driven by connections and disconnections between micro-plates [17, 18]. Such colonisation/expansion events, followed by successive fragmentation episodes, were also associated with the aridification of the climate that began with the last glaciations [18]. Thus, in addition to geographical isolation, the diversification of the Mediterranean flora was strengthened by progressive climatic modifications related to the onset of the Mediterranean climatic regime during the Pliocene (ca. 3.2 kya) [18]. These processes explain the current pattern of endemic *taxa* that are particularly concentrated in stressful habitats, often characterised by a low interspecific competition (e.g. psammophilous and halophytic places and mountain peaks) [8, 11, 19]. Historically, endemic plants in the Mediterranean Basin have also been subjected to intense disturbances, such as deforestation, agriculture, fires, overgrazing, urbanisation, wars and pollution [16, 20–22]. Therefore, studies of the distribution and ecology of endemic plant species at regional and local scales are pivotal for conservation planning.

This research is a representative case study of Mediterranean endemic plants. Indeed, Sardinia is the second largest island of the Mediterranean Basin and it is considered an important centre of plant endemisms [14, 17]. In this paper, we present a regional-scale analysis of Endemic Vascular Plant Richness (EVPR) inside a 1-km resolution grid covering all surfaces of the island. Our main aim was to investigate the ways that topography, geology, climate and human influence have contributed to explain the distribution of EVPR in Sardinia. Additionally, regional EVPR (number of endemic plant *taxa* inside each 100 m elevation interval) was measured to compare, at different scales, EVPR patterns along the elevation gradient.

## Materials and methods

### Study area

Sardinia (Italy) and its ca. 399 satellite minor islands are located in the central part of the western Mediterranean Basin and cover a surface area of around 24,090 km^2^. In the Mediterranean biogeographic region, Sardinia is particularly related to Corsica and the Tuscan Archipelago; together these three areas constitute an independent biogeographical province [23].

**Fig 1:**
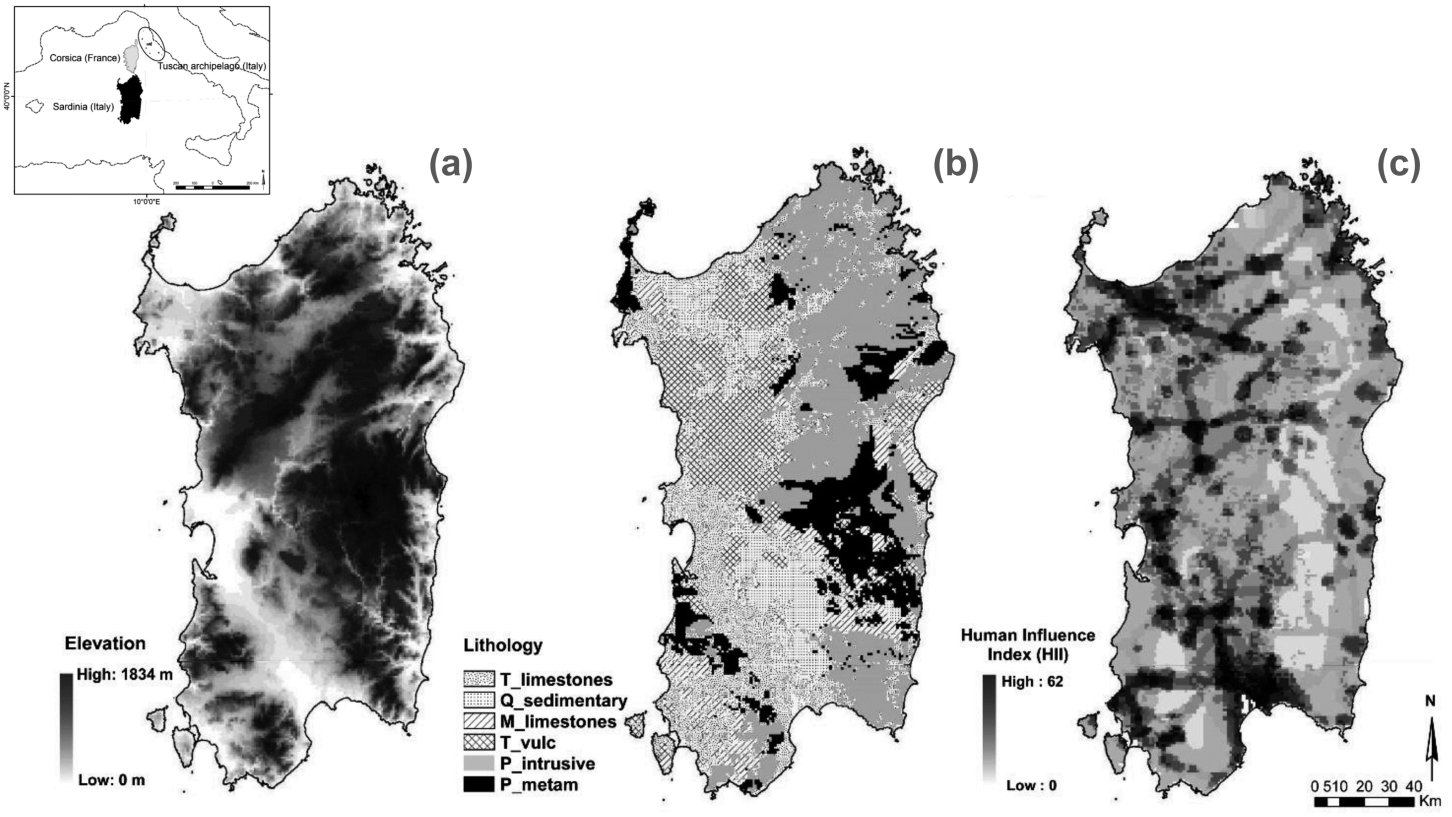
Maps of elevation, topography and Human Influence Index (HII) in Sardinia. Maps on the spatial distribution of (a) elevation, (b) the simplified lithology subdivided into six categories: Quaternary sedimentary outcrops (Q_sedimentary), Tertiary limestone outcrops (T_limestones), Tertiary volcanic outcrops (T_vulc), Mesozoic limestone outcrops (M_limestones), Paleozoic metamorphic outcrops (P_meta) and Paleozoic intrusive outcrops (P_intrusive) and the (c) HII [28].

Sardinia is mainly mountainous (Fig 1A), with several isolated groups of mountains or massifs such as Limbara, Sette Fratelli, Monti del Sulcis, Supramonte and Gennargentu, the highest of them a maximum altitude of 1,834 m, but much of the island is comprised of hilly lands, plateaus and few plains. Its coast is characterised by a variety of landscapes, such as cliffs, sandy dunes and beaches. Substrata and related environments are very heterogeneous, and are mainly composed of Palaeozoic metamorphites and batholiths, a sedimentary lithostratigraphic complex related to a Mesozoic marine transgression, Tertiary marine and volcanic depositions related to the opening of the Tyrrenian Basin (Fig 1B) and Quaternary alluvial deposits [24]. Bioclimatically, two macrobioclimates (Mediterranean pluviseasonal oceanic and Temperate oceanic), four classes of continentality (from weak semihyperoceanic to weak subcontinental), eight thermotypic horizons (from lower thermomediterranean to upper supratemperate) and seven ombrothermic horizons (from lower dry to lower hyperhumid) were identified [25, 26]. The long presence of humans on the island (since the Lower Palaeolithic) has been pivotal in shaping the current landscape [27]. In recent decades, the population in mountain villages has gradually declined, while the largest towns have expanded due to economic development. Lowland plains and coastal zones have also expanded rapidly due to agricultural and touristic development (Fig 1C). This is a common trend among Mediterranean islands, which has caused significant changes in their landscapes [21].

### Floristic data

There are a total of 2,494 *taxa* recognised in Sardinia [29]. This study focuses on the Endemic Vascular Plants (hereafter, EVP), which were identified based on the list in Fenu *et al*. [23]. This list was updated by selecting all the 294 endemic plant *taxa* (total EVP), then in turn sub-dividing these into *taxa* exclusive to Sardinia (187 *taxa*; exclusive EVP), and *taxa* also present in Corsica and the Tuscan Archipelago (107 *taxa*; insular EVP; for details see S1 Table).

The geodatabase of all EVP was assembled from published literature, *Herbarium* collections (CAG, CAT, FI, RO, SASSA, SS, TO) and the authors’ own unpublished field survey records. A total of 60,301 occurrence records were carefully revised to avoid the potential errors due to factors such as approximation of the collection locations or inclusion of extinct localities. Problems related to inhomogeneous sampling efforts were reduced as much as possible by visiting a great part of the territory between 2006 and 2016, paying particular attention to environments similar to those where endemic species were already known to exist, and systematically visiting all phytogeographic subsectors (recently defined on the basis of the same EVP presence/absence [23]).

Subsequently, from the 60,301 EVP occurrence records, we built a 1×1 km grid-based matrix for all Sardinian territory to account for three response variables: (1) the richness of exclusive EVP (hereafter, exclusive EVPR), (2) the richness of insular EVP (insular EVPR) and (3) the richness of all EVP (total EVPR). From the initial number of 36,235 cells, our analyses were restricted to only those grid cells with a minimum of one exclusive (2466), insular (34,375) or total EVP (34,603 cells). This also allowed us to reduce problems related to sampling bias, as all cells used were visited by the authors or by other botanists during recent decades. For the 1 km resolution grid map of the three response variables, see the supporting information (S1 Map).

### Explanatory variables

All explanatory variables used for this study were derived from high-resolution free datasets. A total of 16 predictors were subdivided into three groups: topography and geology (five variables), climate (six variables), and human influence (five variables).

#### Topography and geology

We used two variables, elevation and slope, that are strictly associated with topography and three further variables related to geology: number of geological units, number of land units and lithology. Elevation and slope were computed by averaging values from a 10 m resolution Digital Terrain Model (DTM; available at the institutional Sardinian geoportal, http://www.sardegnageoportale.it). The number of geological units per cell was determined from a 1:25,000 geological map (available from the same institutional Sardinian geoportal), while the number of land units was identified from the "Land Units Map of Italy" [30], kindly provided in raster format by the authors. A land unit was defined as a zone that displays a certain degree of homogeneity according to a uniformity criterion based on lithological and geomorphological traits [30]. Lithology was elaborated by dividing the 1:25,000 geological map above six categories: (1) Quaternary sedimentary outcrops, (2) Tertiary limestone outcrops, (3) Tertiary volcanic outcrops, (4) Mesozoic limestone outcrops, (5) Paleozoic metamorphic outcrops and (6) Paleozoic intrusive outcrops.

#### Climate

Six bioclimatic variables from the WorldClim database version 1.4 (years 1950–2000) [31] with a spatial resolution of 30 arc seconds (~1 km) were used: annual mean temperature (Bio1), minimum temperature of the coldest month (Bio6), annual temperature range (Bio7), annual precipitation (Bio12), precipitation seasonality (Bio15) and precipitation of driest quarter (Bio17).

#### Human influence

We used five variables related to human influence. The first four of them were obtained from the institutional Sardinian geoportal (http://www.sardegnageoportale.it): (1) Roads, calculated as the amount of roads (in km) per grid (from the shapefile of the road network); (2) Number of buildings, calculated from a shape point file obtained by extrapolating local landscape maps; (3) Fires as an index (i) computed from the shapefiles of the burned areas (2005–2013) and taking into account which cells had been affected by fire, and how many times each had experienced fires (i = 0, 1 ≤ i ≤ 9); (4) Land use ratio, computed from the CORINE land use map, which represents the proportion of the area covered by units belonging to the 1–2 Land Use first levels (i.e. anthropogenic uses) against the total surface. High Land use ratio values (i.e. approaching 1:1) were accounted as highly anthropogenic areas, while lower values were considered areas that were more natural. The fifth variable, the (5) Human Influence Index (HII) was obtained from the Wildlife Conservation Society (WCS) and the Centre for International Earth Science Information Network (CIESIN) [28], a free worldwide dataset of 1 km grid cells created from nine global data layers covering human population pressure (population density), human land use and infrastructure (built-up areas, night-time lights, land use/land cover), and human access (coastlines, roads, railroads, navigable rivers).

### Statistical analyses

Methods of variable reduction to avoid collinearity were carried out following Irl *et al*. [10]. First, linear relationships between response and explanatory variables were assessed via bivariate correlations; we used polyserial correlations implemented by the ‘polycor’ R package [32], which enabled us to also include categorical variables. Explanatory variables with correlations -0.1 ≤ r ≤ 0.1 were excluded due to their weak explanatory power [2]. In the second step, collinearity was addressed by testing correlations for each possible pair of explanatory variables. If |r| > 0.7, the explanatory variable performing poorer with the response variable was excluded. Correlation results for all factors are reported in the supporting information (for details see S2 Table). The software GeoDa 1.8.14 [33] was used to verify the absence of spatial autocorrelation in the ordinary least squares residuals with the Lagrange Multiplier (LM) test [34].

The EVPR of all groups of endemics were then fitted by using Generalized Linear Models (GLMs) with Poisson error distribution and log-link function. Likelihood ratio tests based forward selection were applied to check for any significant improvement within models, where variables were included if the related p-value was above 0.05 and removed if the related p-value was above 0.10.

**Fig 2:**
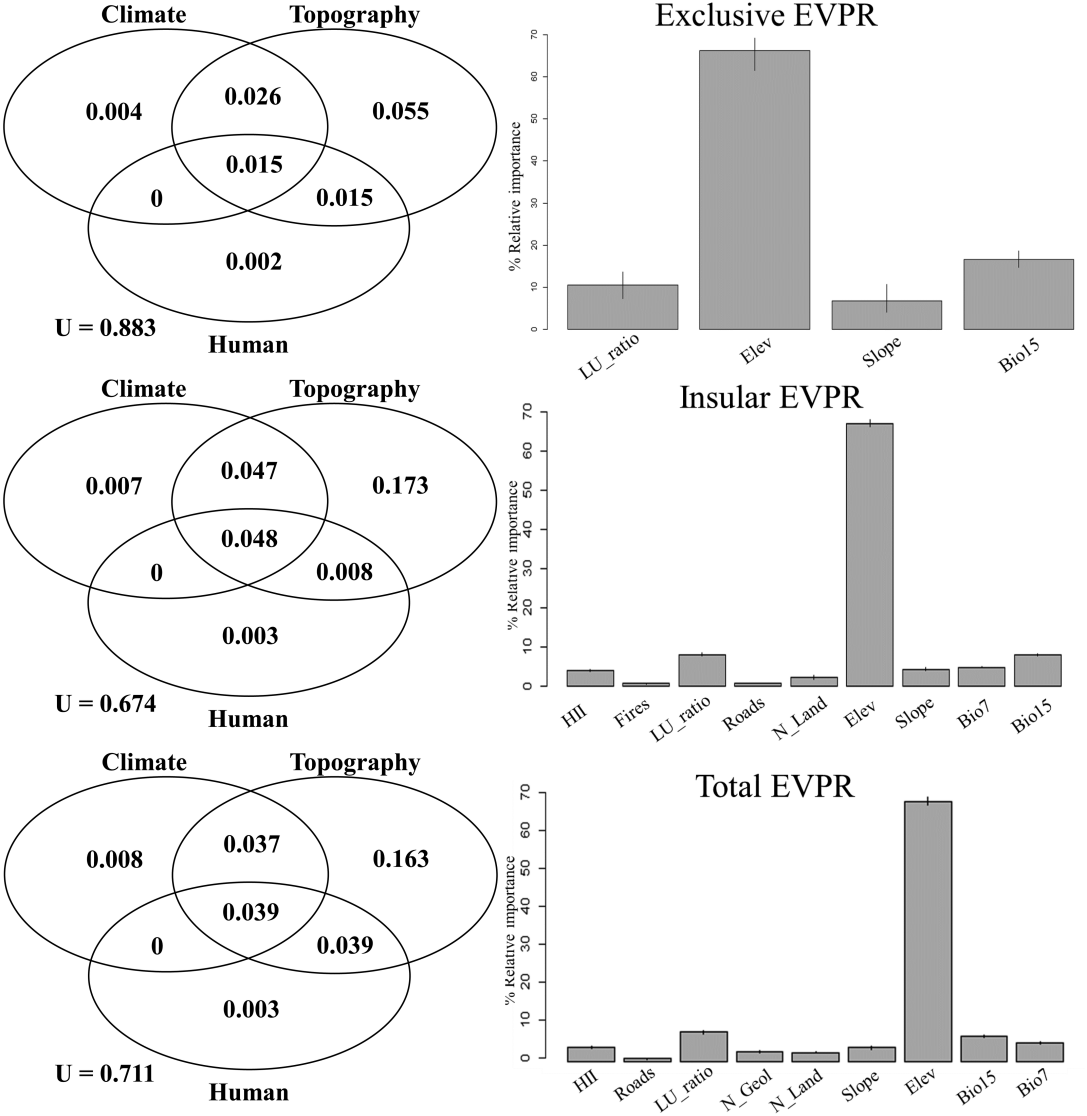
Variance partitioning based on the GLM results for total EVPR, insular EVPR and exclusive EVPR and relative importance of each explanatory variable. The Unexplained (U) and the explained variance of each group of explanatory variables (Human influence (Human), Climate, and Topography and geology (Topography)) are shown on the left. Figures on the right display the relative importance of each explanatory variable calculated as the normalised percentage contribution to the adjusted R^2^ for the respective response variable. See S3 Table for detailed values of the contribution of each group of and singular explanatory variable.

Therefore, variance partitioning for GLMs was implemented to assess the overall importance of climate, topography and human influence [35]. GLMs used for variance partitioning were repeated for each richness group and only included noncorrelated predictors with coefficients of estimates significant at P < 0.05 (Table 1). The variance partitioning approach enabled us to quantify the independent and joint explanatory power of different groups of variables by estimating the proportion of variation from a given response variable that can be attributed exclusively to one set of explanatory variables once the effect of the other explanatory variables has been taken into account [10, 35] (see S3 Table for details). The contribution of each set of variables (i.e. topography, climate or human influence) was based on the amount of deviance it accounted for (D^2^) [36], computed by the *Dsquared* function in the ‘modEvA’ package for R (Fig 2) [37]. For GLMs, D^2^ measures how much deviance a given model explains compared to a model with no variables (the null model) [37].

**Table 1:** Results of generalized linear models (GLMs) showing the set of variables explaining richness of total, insular and exclusive Endemic Vascular Plant Species Richness (EVPR).

Total EVPR					
Variables^a^	Categories^b^	Estimate	z-value	χ2	P^c^
HII	H	-1.71	-3.13	0.2	**<0.001**
LU_Ratio	H	-1.56	-18.02	6.7	**0.009**
Roads	H	-4.29	-11.60	136.2	**<0.001**
N_Land	T	-5.87	-2.58	330.8	**<0.001**
Slope	T	8.48	17.05	293.2	**<0.001**
N_Geol	T	1.00	0.49	9.6	0.625
Elev	T	1.95	101.06	9858.2	**<0.001**
Bio7	C	1.75	9.96	98.8	**<0.001**
Bio15	C	-8.50	-16.52	270.2	**<0.001**
**Insular EVPR**					
HII	H	-2.12	5.63	14.3	**<0.001**
Fires	H	-3.51	-4.67	21.3	**<0.001**
LU_ratio	H	-4.62	-2.16	4.7	**0.031**
Roads	H	-2.62	-2.65	4.2	**<0.01**
N_Land	T	-1.27	-14.24	205.8	**<0.001**
N_Geol	T	-3.05	-1.30	1.7	0.194
Elev	T	1.68	82.59	6594.8	**<0.001**
Slope	T	8.35	16.10	261.5	**<0.001**
Bio7	C	8.85	4.86	122.7	**<0.001**
Bio15	C	-6.01	-11.13	23.6	**<0.001**
**Exclusive EVPR**					
HII	H	-0.04	-0.21	0.04	0.832
LU_ratio	H	-2.91	-3.21	10.51	**<0.001**
Elev	T	8.83	15.57	258.76	**<0.001**
Slope	T	6.66	4.07	16.69	**<0.001**
Bio15	C	2.67	-4.71	22.23	**<0.001**

**Note:** The Poisson distribution with log link function was chosen for all models. Only variables which were not excluded for high collinearity are shown.

^**a**^ Variable abbreviations: HII = Human Influence Index; Fires = index of fires occurred among the years 2005–2013; LU_ratio = ratio of 1–2 Land Use first levels (i.e. anthropogenic uses) and the total surface; Roads = kilometres of roads per grid; N_Geol = number of geological units; N_Land = number of land units; Elev = elevation; Bio7 = annual range of temperature; Bio15 = precipitation seasonality.

^**b**^ H = Human influence; T = Topography and geology; C = Climate

^c^ Significance (in bold for P < 0.05) of the likelihood ratio tests (LRT) was determined using the Chi-Squared (χ2) contribution with 1 degree of freedom

In addition, the percentage of relative importance for each response variable was calculated using hierarchical partitioning of variance, employing the *lmg* metric implemented in the ‘relaimpo’ R package [38]. This procedure has been proposed to decompose the variance of final models among different predictors and interactions and has been widely used in recent ecological studies e.g. [10, 39, 40].

To investigate the specific relationship between elevation and EVPR and a possible interaction between elevation and area, the analysed region was subdivided into 100 m elevation intervals and the variation in number of 1 km grid cells per each interval was plotted and compared with the variation in local (number of endemic plant *taxa* inside each 1 km grid cell) and regional (number of endemic plant *taxa* inside each 100 m elevation interval) EVPR.

## Results

After excluding collinear and weak explanatory predictors, there were eight remaining correlated variables with significant relationships for total EVPR, eight for insular EVPR and four for exclusive EVPR (Table 1). No high spatial autocorrelations were detected in the residuals for total EVPR (LM = 0.71, P = 0.19), insular EVPR (LM = 0.64, P = 0.26) or exclusive EVPR (LM = 0.22, P = 0.64).

All predictors related to human influence, the number of land units, and precipitation seasonality (Bio15), demonstrated a negative correlation with all groups of EVPR. On the contrary, EVPR increased with elevation, slope and annual temperature ranges (Bio7).

In every case, elevation alone accounted for more variance than all other variables together (Fig 2; see S4 Table for details). Accordingly, most variation in exclusive, insular and total local EVPR was explained by the topography and geology subsets, followed by climate, and by human influence (Fig 2). All shared variances were lower than the variances of topography and geology subsets alone, while shared variances between topography and geology and climate and human influence subsets were higher than the independent variances explained by the last two subsets. Nonetheless, a moderately large amount of variance remained unexplained; this was more evident for the exclusive EVPR (88.3%) than for total and insular EVPR (71.1% and 67.4%, respectively).

**Fig 3:**
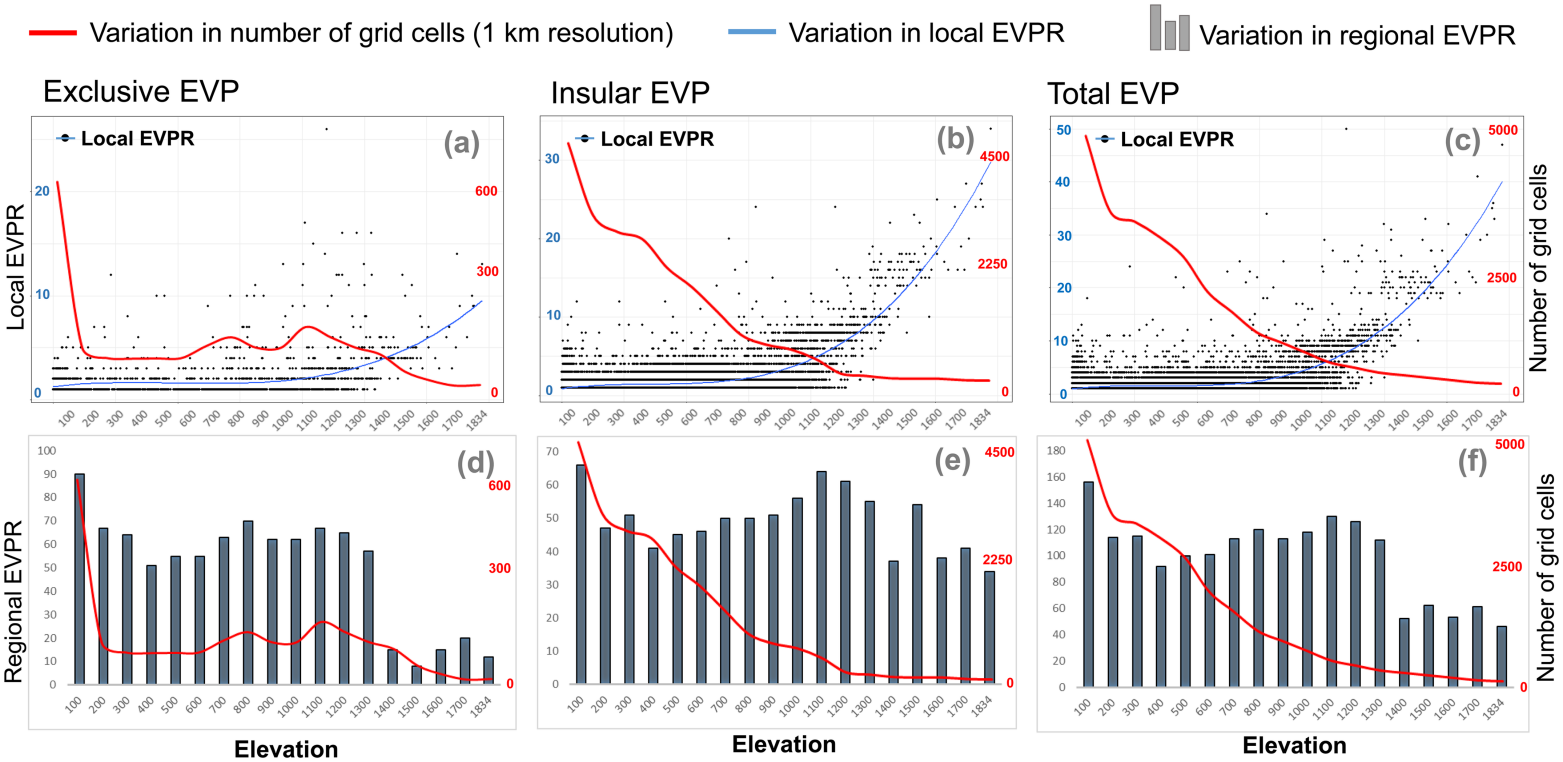
Variations in EVPR of exclusive EVP, insular EVP and total EVP. Variations in local EVPR (number of endemic plant *taxa* inside each 1 km grid cell) of exclusive EVP (a; N = 2466), insular EVP (b; N = 34375) and total EVP (c; N = 34603) are shown in the upper part of the figure. Variations in regional EVPR (number of endemic plant *taxa* inside each 100 m elevation interval) of exclusive EVP (d), insular EVP (e) and total EVP (f) are shown in the lower part of the figure. Variations in the area of territory considered (number of 1 km resolution grid cells at each elevation interval) were superimposed on both local and regional EVPR.

Comparisons among variations in exclusive, insular and total EVPR and elevation stressed that the three EVP groups showed similar exponential patterns with the highest local EVPR in cells at the highest elevations (approx. > 1300 m a.s.l.) (Fig 3A, 3B and 3C). This pattern was similar among the three analysed groups (exclusive, insular and total), although it was more evident for insular than for exclusive EVPR (Fig 3A and 3B). In contrast, the regional EVPR, especially of exclusive EVP (Fig 3D), decrease with altitude partly due to the decreasing area covered along altitude (i.e. the number of 1 km grid cells per elevation interval) (Fig 3D, 3E and 3F). This was less evident for insular EVP and, consequently, for total EVP, the richness of which was more constant along the elevation gradient.

## Discussion

As previously found for other Mediterranean continental islands [3, 11, 17], and also for the highest mountain area of Sardinia [14], elevation revealed to be a crucial factor in explaining EVPR. In particular, the highest EVPR was found at the highest elevations. These results support authors who previously highlighted the mountainous Gennargenteo and Supramontano biogeographic sectors as important areas for the conservation of both plant diversity [14, 23, 41] and other organisms, such as bats, butterflies and amphibians [42–44].

Considering that high elevations comprise smaller areas, high EVPR are also reflecting high EVP concentrations. On the other hand, the area *per se* hypothesis, which assumes that species’ richness increases with area [13], counterbalances the increase in EVPR in restricted areas at higher altitudes. Indeed, when regional EVPR is considered, the number of exclusive EVP in particular increases with area (i.e. number of 1 km grid cells) and decreases with the EVP concentration (i.e. local EVPR) and elevation, suggesting that EVPR patterns might be influenced by the interaction between the area and elevation. For instance, besides their large number, endemic *taxa* along the coast (i.e. first 100-m elevation interval) are less concentrated than in mountains, occupying many small places with particular morphologies and high degrees of isolation (e.g. small islets and cliffs) [41, 45].

The increase in endemic plant species’ richness as elevation increases was also found in other Mediterranean contexts [9, 11, 46, 47]; otherwise, other research reported an increase in the endemic plant species richness at intermediate altitudes in islands with mountain systems reaching elevations of more than 2000 m a.s.l. [3, 48]. In our case, an increase in EVPR at mid elevation ranges is likely to be more evident at larger scales (i.e. when data is pooled across 100 m intervals) than at smaller scales (i.e. when data is pooled inside 1 km grid cells), suggesting that the relationship between elevation and EVPR might be also sensitive to the sampling size.

Species composition, and the richness in the most interesting areas of endemisms (mainly mountainous areas, but also some coastal areas, such as small islets and cliffs), were also related to the ancient traditional land use of ecosystems [27], characterised by the exploitation of lowlands, leaving the higher slopes and elevations for less intensive touristic and agro-pastoral uses. Our results regarding the negative relationship between EVPR (exclusive EVPR in particular) and the proportion of the area covered by units affected by anthropogenic uses (LU_ratio) partially confirmed the widespread idea that humans, with their accompanying land uses changes, acted as major extinction filters [1, 12, 49].

Relationships between EVPR and elevation might be influenced by other important factors, mainly climate, the effects of which might be partly masked by elevation or were not measurable. According to previous researches e.g. [3, 6, 8, 10], plant diversity patterns result from the interaction or addition of multiple biogeographic and ecological factors, the effect of which is more or less evident depending on the scale of observation [50]. At local scale, the negative relationship between EVPR and precipitation seasonality (Bio15), and the positive one with the annual temperature range (Bio7) suggest a possible correlation between these variables and elevation, a trend that is quite common in other areas [51] and which has, in our case, a moderate collinearity. On the other hand, precipitation and temperatures are *per se* crucial factors in plant species richness [6, 14] and evolution [17–18], and their importance has been also underlined from a conservation perspective, especially related to climate changes [5, 52, 53]. Since an increase in precipitation seasonality is expected to be in the Mediterranean Basin under climate change [54], conservation efforts should be focused in Sardinia on endemic plants with specific moisture requirements, such as the already endangered *Pinguicula sehuensis* Bacch., Cannas & Peruzzi, *Ribes sardoum* Martelli and all species of the genus *Aquilegia* [55–57].

Local EVPR was significantly influenced by variables, such as the slope and the number of land units, which are often in synergy with elevation [19, 47]. For instance, both elevation and slope play important roles in increasing the degree of isolation in terms of dispersal events and human colonization [3, 47]. First, it has been documented that endemic species richness usually peaks at higher elevation and rocky places than total species richness; this could be related to the increasing isolation and the decreasing surface area of high mountain regions, leading to speciation events in small and fragmented species populations [3, 58]. In addition, habitats, such as mountain grasslands, rocky habitats as well as psammophilous and halophytic places, with a high degree of stress and where vegetation and competition are low, are characterized by the frequent occurrence of endemics.

Although it is common to find a positive effect of habitat diversity on EVPR at regional scale by increasing space available for niche partitioning and speciation and, thus, for species coexistence [10], discrepancies were also previously found between regional and local scale analyses [50]. For instance, our results at 1 km spatial resolution showed a negative correlation of land unit diversity with EVPR, suggesting that, at local scale, land unit diversity reflects the negative effect of habitat fragmentation instead of the common positive influence of habitat diversity. The weak and insignificant relationships between, respectively, insular and exclusive EVPR and human influence could be interpreted as an ostensible lack of human threats to EVP; however, further considerations should be weighed. Firstly, analyses of human presence as a determining factor of current Mediterranean landscape and biodiversity patterns have faced several shortcomings, principally related to difficulties in accurately evaluating consequences of such a long-term presence, as well as the many indirect factors triggered by it. Therefore, if present-day biodiversity should be biased toward species that are generally more tolerant of humans [1], analyses accounting for absences or extinction events which can be related with certainty to human influence are barely feasible [21].

Assuming that richness of endemisms reflects species speciation rate reasonably well [11, 46], the positive influence of the slope and elevation predictors could also suggest an increase in speciation rate associated with topography-driven isolation [46, 47]. Although it is not directly measurable by our analyses, this is more evident at fine scale (i.e. when data is pooled inside 1 km grid cells) and for spread than for exclusive EVP. The latter suggests that the pattern of insular EVP is more related to the geographical speciation promoted by the alternation of glacial and warm interglacial phases than the pattern of exclusive EVP. This could be related to findings obtained by comparing island and mainland mountain systems in the Mediterranean Basin e.g. [11, 14, 59, 60], which showed, in some cases, a weaker endemic species richness-elevation relationship for islands. Indeed, the same isolation that can facilitate the speciation of EVP, especially of exclusive EVP [11], may, at the same time, limit the ingression and speciation of EVP related to the alternation of glacial and interglacial phases [17, 61].

For the first time, our study provides a general-picture, from the lowest point on the coast up to the highest mountain peaks, about the distribution patterns of all endemic vascular flora of Sardinia. Nonetheless, a large section of variance remains unexplained, mainly because the distribution of EVPR can hardly be related to all possible past and present causes. Since the relationship between EVPR and elevation might be sensitive to the sampling grain, a possible way to improve the knowledge in this field could be to compare analyses and results at different resolutions by considering the same parameters inside both coarser or, if possible, finer grids. Alternatively, specific local studies or, despite their costs, species-specific empirical researches may be the only feasible approach for understanding some specific issues.

## Supporting information

S1 Table Checklist of Endemic Vascular Plants (EVP) used for this study (DOCX)

S2 Table Bivariate correlation matrix of Endemic Vascular Plant Species Richness (EVPR) with all explanatory variables and of explanatory variables with each other. (DOCX)

S3 Table Fractions of variation explained by climate, topography and human factors. (DOCX)

S4 Table Total and proportion of variance explained by each explanatory variable. (DOCX)

S1 Map 1-km resolution grid cells of exclusive EVPR, insular EVPR and total EVPR. (ZIP)
