# Supplementary material for: Disentangling the influence of environmental and anthropogenic factors on the distribution of endemic vascular plants in Sardinia
Source: PLoS One. 2017 Aug 2;12(8):e0182539. doi: 10.1371/journal.pone.0182539 (PMC5540478; doi:10.1371/journal.pone.0182539)
Supplement: S1 Table — (DOCX) [file pone.0182539.s001.docx]

**S1 Table. Checklist of Endemic Vascular Plants (EVP) used for this study**

List endemics exclusive to Sardinia (exclusive EVP) and endemics to Sardinia, Corsica and Tuscan Archipelago islands (insular EVP). The nomenclature and status of the *taxa* included in the study are based on Fenu et al. (2014, updated).

| **Scientific name** | **Family** | **Endemic status** |
| --- | --- | --- |
| *Allium parciflorum* Viv. | Alliaceae | insular EVP |
| *Allium roseum* var. *insulare* Gennari | Alliaceae | insular EVP |
| *Alyssum tavolarae* Briq. | Brassicaceae | exclusive EVP |
| *Anarrhinum corsicum* Jordan *et* Fourr. | Plantaginaceae | insular EVP |
| *Anchusa capellii* Moris | Boraginaceae | exclusive EVP |
| *Anchusa crispa* Viv. ssp. *crispa* | Boraginaceae | insular EVP |
| *Anchusa crispa* Viv. ssp. *maritima* (Vals.) Selvi *et* Bigazzi | Boraginaceae | exclusive EVP |
| *Anchusa formosa* Selvi, Bigazzi *et* Bacch. | Boraginaceae | exclusive EVP |
| *Anchusa littorea* Moris | Boraginaceae | exclusive EVP |
| *Anchusa montelinasana*Angius, Pontecorvo *et* Selvi *ex* Bacch., Coppi, Pontecorvo *et* Selvi | Boraginaceae | exclusive EVP |
| *Anchusa sardoa* (Illario) Selvi *et* Bigazzi | Boraginaceae | exclusive EVP |
| *Anthyllis hermanniae* L. ssp. *ichnusae* Brullo *et* Giusso | Fabaceae | exclusive EVP |
| *Aquilegia barbaricina* Arrigoni *et* E. Nardi | Ranunculaceae | exclusive EVP |
| *Aquilegia cremnophila* Bacch., Brullo, Congiu, Fenu, J. Garrido *et* Mattana | Ranunculaceae | exclusive EVP |
| *Aquilegia nugorensis* Arrigoni *et* E. Nardi | Ranunculaceae | exclusive EVP |
| *Aquilegia nuragica* Arrigoni *et* E. Nardi | Ranunculaceae | exclusive EVP |
| *Aristolochia rotunda* L. ssp. *insularis* Nardi *et* Arrigoni | Aristolochiaceae | insular EVP |
| *Aristolochia tyrrhena* Nardi *et* Arrigoni | Aristolochiaceae | exclusive EVP |
| *Armeria morisii* Boiss. *in* A. DC. | Plumbaginaceae | exclusive EVP |
| *Armeria sardoa* Spreng. ssp. *genargentea* Arrigoni | Plumbaginaceae | exclusive EVP |
| *Armeria sardoa* Spreng. ssp. *sardoa* | Plumbaginaceae | exclusive EVP |
| *Armeria sulcitana* Arrigoni | Plumbaginaceae | exclusive EVP |
| *Artemisia gallica* Willd. ssp. *densiflora* (Viv.) Gamisans | Asteraceae | insular EVP |
| *Arum pictum* L. *fil.* ssp. *pictum* | Araceae | insular EVP |
| *Asperula deficens* Viv. | Rubiaceae | exclusive EVP |
| *Asperula pumila* Moris | Rubiaceae | exclusive EVP |
| *Asplenium* x *cyrnosardoum* Rasbach, Vida *et* Reichst. | Aspleniaceae | insular EVP |
| *Astragalus genargenteus* Moris | Fabaceae | exclusive EVP |
| *Astragalus gennarii* Bacch. *et* Brullo | Fabaceae | exclusive EVP |
| *Astragalus maritimus* Moris | Fabaceae | exclusive EVP |
| *Astragalus tegulensis* Bacch. *et* Brullo | Fabaceae | exclusive EVP |
| *Astragalus terraccianoi* Vals. | Fabaceae | insular EVP |
| *Astragalus thermensis* Vals. | Fabaceae | exclusive EVP |
| *Astragalus verrucosus* Moris | Fabaceae | exclusive EVP |
| *Barbarea rupicola* Moris | Brassicaceae | insular EVP |
| *Bellium crassifolium* Moris | Asteraceae | exclusive EVP |
| *Bellium crassifolium* Moris var. *canescens* Gennari | Asteraceae | exclusive EVP |
| *Biscutella morisiana* Raffaelli | Brassicaceae | insular EVP |
| *Borago morisiana* Bigazzi *et* Ricceri | Boraginaceae | exclusive EVP |
| *Borago pygmaea* (DC.) Chater *et* Greuter | Boraginaceae | insular EVP |
| *Brassica tyrrhena* Giotta, Piccitto *et* Arrigoni | Brassicaceae | exclusive EVP |
| *Bryonia marmorata* Petit | Cucurbitaceae | insular EVP |
| *Bunium corydalinum* DC. ssp. *corydalinum* | Apiaceae | insular EVP |
| *Buphthalmum inuloides* Moris | Asteraceae | exclusive EVP |
| *Campanula forsythii* (Arcangeli) Podlech | Campanulaceae | exclusive EVP |
| *Carduus fasciculiflorus* Viv. | Asteraceae | insular EVP |
| *Carex microcarpa* Bertol. *ex* Moris | Cyperaceae | insular EVP |
| *Carlina macrocephala* ssp. *macrocephala* Moris | Asteraceae | insular EVP |
| *Centaurea corensis* Vals. *et* Filigh. | Asteraceae | exclusive EVP |
| *Centaurea filiformis* Viv. ssp. *ferulacea* (Martelli) Arrigoni | Asteraceae | exclusive EVP |
| *Centaurea filiformis* Viv. ssp. *filiformis* | Asteraceae | exclusive EVP |
| *Centaurea forsythiana* Lev. *pro hybr.* | Asteraceae | exclusive EVP |
| *Centaurea horrida* Badarò | Asteraceae | exclusive EVP |
| *Centaurea magistrorum* Arrigoni *et* Camarda | Asteraceae | exclusive EVP |
| *Centaurium erythraea* Rafn ssp. *rhodense* (Boiss. *et* Reut.) Melderis var. *sanguineum* (Mabille) Gamisans | Gentianaceae | insular EVP |
| *Centranthus amazonum* Fridl. *et* A. Raynal | Caprifoliaceae | exclusive EVP |
| *Cephalaria bigazzii* Bacch., Brullo *et* Giusso | Dipsacaceae | exclusive EVP |
| *Cephalaria mediterranea* (Viv.) Szabò | Dipsacaceae | exclusive EVP |
| *Cerastium palustre* Moris | Caryophyllaceae | exclusive EVP |
| *Cerastium supramontanum* Arrigoni | Caryophyllaceae | exclusive EVP |
| *Charybdis glaucophylla* Bacch., Brullo, D’Emerico, Pontec. *et* Salmeri | Asparagaceae | exclusive EVP |
| *Cistus creticus* L. var. *corsicus* (Loisel.) Greuter | Cistaceae | insular EVP |
| *Clinopodium* *sandalioticum* (Bacch. *et* Brullo) Bacch. *et* Brullo *ex* Peruzzi *et* F. Conti | Lamiaceae | exclusive EVP |
| *Clinopodium sardoum* (Asch. *et* Levier) Peruzzi *et* F. Conti | Lamiaceae | exclusive EVP |
| *Coincya monensis* (L.) Greuter *et* Burdet ssp. *recurvata* (All.) E.A. Leadlay | Brassicaceae | insular EVP |
| *Colchicum arenasii* Fridl. | Colchicaceae | exclusive EVP |
| *Colchicum verlaqueae* Fridl. | Colchicaceae | exclusive EVP |
| *Colchium gonarei* Camarda | Colchicaceae | exclusive EVP |
| *Crepis caespitosa* Gren. *et* Godr. | Asteraceae | insular EVP |
| *Crocus minimus* DC. *in* Rèdouté | Iridaceae | insular EVP |
| *Cuscuta epithynum* (L.) L. ssp. *corsicana* (Junk.) J. Lambinon | Convolvulaceae | insular EVP |
| *Cymbalaria muelleri* (Moris) A. Chev. | Plantaginaceae | exclusive EVP |
| *Cynoglossum barbaricinum* Arrigoni *et* Selvi | Boraginaceae | exclusive EVP |
| *Delphinium longipes* Moris | Ranunculaceae | exclusive EVP |
| *Dianthus cyathophorus* Moris | Caryophyllaceae | exclusive EVP |
| *Dianthus genargenteus* Bacch., Brullo, Casti *et* Giusso | Caryophyllaceae | exclusive EVP |
| *Dianthus ichnusae* Bacch., Brullo, Casti *et* Giusso ssp. *ichnusae* | Caryophyllaceae | exclusive EVP |
| *Dianthus ichnusae* Bacch., Brullo, Casti et Giusso ssp. *toddei* Bacch., Brullo, Casti *et* Giusso | Caryophyllaceae | exclusive EVP |
| *Dianthus insularis* Bacch., Brullo, Casti *et* Giusso | Caryophyllaceae | exclusive EVP |
| *Dianthus morisianus* Vals. | Caryophyllaceae | exclusive EVP |
| *Dianthus mossanus* Bacch. *et* Brullo | Caryophyllaceae | exclusive EVP |
| *Dianthus oliastrae* Bacch., Brullo, Casti *et* Giusso | Caryophyllaceae | exclusive EVP |
| *Dianthus sardous* Bacch., Brullo, Casti *et* Giusso | Caryophyllaceae | exclusive EVP |
| *Digitalis purpurea* L. var. *gyspergerae* (Rouy) Fiori | Plantaginaceae | insular EVP |
| *Dipsacus ferox* Loisel. | Dipsacaceae | insular EVP |
| *Dipsacus valsecchii* Camarda | Dipsacaceae | exclusive EVP |
| *Echium anchusoides* Bacch., Brullo *et* Selvi | Boraginaceae | exclusive EVP |
| *Elymus acutus* (DC.) Thiébaud | Poaceae | insular EVP |
| *Elymus* *corsicus* (Hackel) Kerguélen | Poaceae | insular EVP |
| *Erodium corsicum* Léman *in* Lam. *et* DC. | Geraniaceae | insular EVP |
| *Euphorbia amygdaloides* L. ssp. *semiperfoliata* (Viv.) Radcl.-Sm. | Euphorbiaceae | insular EVP |
| *Euphrasia nana* (Rouy) Prain | Orobanchaceae | insular EVP |
| *Ferula arrigonii* Bocchieri | Apiaceae | insular EVP |
| *Festuca alfrediana* Foggi *et* Signorini ssp. *alfrediana* | Poaceae | exclusive EVP |
| *Festuca morisiana* Parl. | Poaceae | exclusive EVP |
| *Festuca sardoa* (Haeckel *in* Barbey) Richter | Poaceae | insular EVP |
| *Filago tyrrhenica* Chrtek *et* Holub *ex* Soldano *et* F. Conti | Asteraceae | insular EVP |
| *Galium corsicum* Spreng. | Rubiaceae | insular EVP |
| *Galium glaucophyllum* Em. Schmid | Rubiaceae | exclusive EVP |
| *Galium schmidii* Arrigoni | Rubiaceae | exclusive EVP |
| *Genista arbusensis* Vals. | Fabaceae | exclusive EVP |
| *Genista bocchierii* Bacch., Feoli Chiapella *et* Brullo | Fabaceae | exclusive EVP |
| *Genista cadasonensis* Vals. | Fabaceae | exclusive EVP |
| *Genista corsica* (Loisel.) DC. *in* Lam. *et* DC. | Fabaceae | insular EVP |
| *Genista ephedroides* DC. | Fabaceae | insular EVP |
| *Genista insularis* Bacch., Feoli Chiapella *et* Brullo ssp. *fodinae* Bacch., Feoli Chiapella *et* Brullo | Fabaceae | exclusive EVP |
| *Genista insularis* Bacch., Feoli Chiapella *et* Brullo ssp. *insularis* | Fabaceae | exclusive EVP |
| *Genista morisii* Colla | Fabaceae | exclusive EVP |
| *Genista ovina* Bacch., Feoli Chiapella *et* Brullo | Fabaceae | exclusive EVP |
| *Genista pichi-sermolliana* Vals. | Fabaceae | exclusive EVP |
| *Genista salzmannii* DC. var. *salzmannii* | Fabaceae | insular EVP |
| *Genista sardoa* Vals. | Fabaceae | exclusive EVP |
| *Genista sulcitana* Vals*.* | Fabaceae | exclusive EVP |
| *Genista toluensis* Vals. | Fabaceae | exclusive EVP |
| *Genista valsecchiae* Brullo *et* De Marco | Fabaceae | exclusive EVP |
| *Glechoma sardoa* (Bég.) Bég. | Lamiaceae | exclusive EVP |
| *Helianthemum morisianum* Bertol. | Cistaceae | exclusive EVP |
| *Helichrysum frigidum* (Labill.) Willd. | Asteraceae | exclusive EVP |
| *Helichrysum montelinasanum* Em. Schmid. | Asteraceae | exclusive EVP |
| *Helichrysum saxatile* Moris ssp. *morisianum* Bacch., Brullo *et* Mossa | Asteraceae | exclusive EVP |
| *Helichrysum saxatile* Moris ssp. *saxatile* | Asteraceae | exclusive EVP |
| *Helleborus lividus* Aiton ssp. *corsicus* (Briq.) P.F. Yeo | Ranunculaceae | insular EVP |
| *Herniaria latifolia* Lapeyr. ssp. *litardierei* Gamisans | Caryophyllaceae | insular EVP |
| *Hieracium bernardii* Rouy | Asteraceae | insular EVP |
| *Hieracium iolai* Arrigoni | Asteraceae | exclusive EVP |
| *Hieracium soleirolianum* Arv. Touv. *et* Briq. | Asteraceae | insular EVP |
| *Hypericum annulatum* Moris | Hypericaceae | exclusive EVP |
| *Hypericum corsicum* Godr. | Hypericaceae | insular EVP |
| *Hypericum hircinum* L. ssp. *hircinum* | Hypericaceae | insular EVP |
| *Hypericum scruglii* Bacch., Brullo *et* Salmeri | Hypericaceae | exclusive EVP |
| *Hypochaeris sardoa* Bacch., Brullo *et* Terrasi | Asteraceae | exclusive EVP |
| *Iberis integerrima* Moris | Brassicaceae | exclusive EVP |
| *Juniperus nana* Willd. var. *corsicana* Lebreton, Mossa *et* Gallet *nom. nud.* | Cupressaceae | insular EVP |
| *Lactuca longidentata* Moris | Asteraceae | exclusive EVP |
| *Lamium garganicum* L. ssp. *corsicum* (Godr. *et* Gren.) Arcang. | Lamiaceae | insular EVP |
| *Lamyropsis microcephala* (Moris) Dittrich *et* Greuter | Asteraceae | exclusive EVP |
| *Lavatera plazzae* Atzei | Malvaceae | exclusive EVP |
| *Lavatera triloba* L. ssp. *pallescens* (Moris) Nyman | Malvaceae | exclusive EVP |
| *Leucojum roseum* Martin | Alliaceae | insular EVP |
| *Limonium acutifolium* (Rchb.) Salmon | Plumbaginaceae | exclusive EVP |
| *Limonium acutifolium* (Rchb.) Salmon ssp. *nymphaeum* (Erben) Arrigoni | Plumbaginaceae | exclusive EVP |
| *Limonium ampuriense* Arrigoni *et* Diana | Plumbaginaceae | exclusive EVP |
| *Limonium articulatum* (Loisel.) Kuntze | Plumbaginaceae | insular EVP |
| *Limonium bosanum* Arrigoni *et* Diana | Plumbaginaceae | exclusive EVP |
| *Limonium capitis-eliae* Erben | Plumbaginaceae | exclusive EVP |
| *Limonium capitis-marci* Arrigoni *et* Diana | Plumbaginaceae | exclusive EVP |
| *Limonium caralitanum* Erben | Plumbaginaceae | exclusive EVP |
| *Limonium carisae* Erben | Plumbaginaceae | exclusive EVP |
| *Limonium contortirameum* (Mabille) Erben | Plumbaginaceae | insular EVP |
| *Limonium coralliforme* Mayer | Plumbaginaceae | exclusive EVP |
| *Limonium cornusianum* Arrigoni *et* Diana | Plumbaginaceae | exclusive EVP |
| *Limonium cunicularium* Arrigoni *et* Diana | Plumbaginaceae | exclusive EVP |
| *Limonium gallurense* Arrigoni *et* Diana | Plumbaginaceae | exclusive EVP |
| *Limonium glomeratum* (Tausch) Erben | Plumbaginaceae | exclusive EVP |
| *Limonium hermaeum* Pignatti | Plumbaginaceae | exclusive EVP |
| *Limonium insulare* (Bég. *et* Landi) Arrigoni *et* Diana | Plumbaginaceae | exclusive EVP |
| *Limonium laetum* (Nyman) Pignatti | Plumbaginaceae | exclusive EVP |
| *Limonium lausianum* Pignatti | Plumbaginaceae | exclusive EVP |
| *Limonium malfatanicum* Erben | Plumbaginaceae | exclusive EVP |
| *Limonium merxmuelleri* Erben | Plumbaginaceae | exclusive EVP |
| *Limonium morisianum* Arrigoni | Plumbaginaceae | exclusive EVP |
| *Limonium multifurcatum* Erben | Plumbaginaceae | exclusive EVP |
| *Limonium oristanum* Mayer | Plumbaginaceae | exclusive EVP |
| *Limonium protohermaeum* Arrigoni *et* Diana | Plumbaginaceae | exclusive EVP |
| *Limonium pseudolaetum* Arrigoni *et* Diana | Plumbaginaceae | exclusive EVP |
| *Limonium pulviniforme* Arrigoni *et* Diana | Plumbaginaceae | exclusive EVP |
| *Limonium racemosum* (Lojac.) Diana | Plumbaginaceae | exclusive EVP |
| *Limonium retirameum* Greuter *et* Burdet | Plumbaginaceae | exclusive EVP |
| *Limonium strictissimum* (Salzmann) Arrigoni | Plumbaginaceae | insular EVP |
| *Limonium sulcitanum* Arrigoni | Plumbaginaceae | exclusive EVP |
| *Limonium tenuifolium* (Bertol. *ex* Moris) Erben | Plumbaginaceae | exclusive EVP |
| *Limonium tharrosianum* Arrigoni *et* Diana | Plumbaginaceae | exclusive EVP |
| *Limonium tibulatium* Pignatti | Plumbaginaceae | exclusive EVP |
| *Limonium tigulianum* Arrigoni *et* Diana | Plumbaginaceae | exclusive EVP |
| *Limonium tyrrhenicum* Arrigoni *et* Diana | Plumbaginaceae | exclusive EVP |
| *Limonium ursanum* Erben | Plumbaginaceae | exclusive EVP |
| *Limonium viniolae* Arrigoni *et* Diana | Plumbaginaceae | exclusive EVP |
| *Linaria arcusangeli* Atzei *et* Camarda | Plantaginaceae | exclusive EVP |
| *Linaria flava* (Poir.) Desf. ssp. *sardoa* (Sommier) A. Terracc. | Plantaginaceae | insular EVP |
| *Linum muelleri* Moris | Linaceae | exclusive EVP |
| *Lotus cytisoides* L. ssp. *conradiae* Gamisans | Fabaceae | insular EVP |
| *Luzula spicata* (L.) DC. ssp. *italica* (Parl.) Arcangeli | Juncaceae | insular EVP |
| *Medicago intertexta* (L.) Mill. var. *tuberculata* Moris | Fabaceae | exclusive EVP |
| *Mentha requienii* Benth. ssp. *requienii* | Lamiaceae | insular EVP |
| *Mercurialis corsica* Cosson | Euphorbiaceae | insular EVP |
| *Micromeria cordata* (Moris *et* Bertol.) Moris | Lamiaceae | exclusive EVP |
| *Morisia monanthos* (Viv.) Asch. *ex* Barbey | Brassicaceae | insular EVP |
| *Myosotis soleirolii* Godr. *in* Gren. *et* Godr. | Boraginaceae | insular EVP |
| *Nananthea perpusilla* (Loisel.) DC. | Asteraceae | insular EVP |
| *Narcissus supramontanus* Arrigoni ssp. *cunicularium* Arrigoni | Amaryllidaceae | exclusive EVP |
| *Narcissus supramontanus* Arrigoni ssp. *supramontanus* | Amaryllidaceae | exclusive EVP |
| *Nepeta foliosa* Moris | Lamiaceae | exclusive EVP |
| *Odontites corsicus* (Loisel.) G. Don *fil.* | Orobanchaceae | insular EVP |
| *Oenanthe lisae* Moris | Apiaceae | exclusive EVP |
| *Ophrys annae* Devillers-Terschuren | Orchidaceae | insular EVP |
| *Ophrys chestermanii* (Wood) Gölz *et* Reinhard | Orchidaceae | exclusive EVP |
| *Ophrys conradiae* Melki *et* Deschâtres | Orchidaceae | insular EVP |
| *Ophrys funerea* Viv. | Orchidaceae | insular EVP |
| *Ophrys morisii* (Martelli) Soo | Orchidaceae | insular EVP |
| *Ophrys normanii* J.J. Wood *pro hybr.* | Orchidaceae | exclusive EVP |
| *Ophrys ortuabis* Grasso *et* Manca | Orchidaceae | exclusive EVP |
| *Ophrys panattensis* Scrugli, Cogoni *et* Pessei | Orchidaceae | exclusive EVP |
| *Ophrys scolopax* Cav. ssp. *sardoa* H. Baumann, Giotta, Lorenz, Künkeke *et* Piccitto | Orchidaceae | exclusive EVP |
| *Ophrys sphegodes* Mill. ssp. *praecox* Corrias | Orchidaceae | insular EVP |
| *Orchis mascula* (L.) L. ssp. *ichnusae* Corrias | Orchidaceae | exclusive EVP |
| *Orchis sardoa* (Scrugli *et* Grasso) Scrugli, Musacchio, D'Emerico, Pellegrino *et* Cozzolino *pro hybr.* | Orchidaceae | exclusive EVP |
| *Ornithogalum corsicum* Jord. *et* Fourr. | Asparagaceae | insular EVP |
| *Orobanche australis* Moris *ex* Bert. | Orobanchaceae | exclusive EVP |
| *Orobanche denudata* Moris | Orobanchaceae | exclusive EVP |
| *Orobanche rapum-genistae* Thuill. ssp. *rigens* Loisel. | Orobanchaceae | insular EVP |
| *Paeonia corsica* Sieber *ex* Tausch | Paeoniaceae | insular EVP |
| *Pancratium illyricum* L. | Alliaceae | insular EVP |
| *Pastinaca sativa* L. ssp. *divaricata* (Desf.) Rouy *et* Camus | Apiaceae | insular EVP |
| *Petrorhagia saxifraga* (L.) Link ssp. *bicolor* (Jord. *et* Fourr.) Gamisans | Caryophyllaceae | insular EVP |
| *Phalaris rotgesii* Fouc. *et* Mandon *ex* Husnot | Poaceae | insular EVP |
| *Phleum sardoum* (Hackel) Hackel *in* Franchet | Poaceae | exclusive EVP |
| *Pinguicula sehuensis* Bacch., Cannas & Peruzzi | Lentibulariaceae | exclusive EVP |
| *Plagius flosculosus* (L.) Alavi *et* Heywood | Asteraceae | insular EVP |
| *Plantago sarda* C.Presl. | Plantaginaceae | insular EVP |
| *Platanthera kuenkelei* H.Bauman subsp. *kuenkelei* var. *sardoa* R. Lorenz, Akhalkatsi, H. Baumann, Cortis, Cogoni et Scrugli | Orchidaceae | exclusive EVP |
| *Poa balbisii* Parl. | Poaceae | insular EVP |
| *Polygala sardoa* Chodat | Polygalaceae | exclusive EVP |
| *Polygala sinisica* Arrigoni | Polygalaceae | exclusive EVP |
| *Polygonum scoparium* Req. *ex* Loisel. | Polygonaceae | insular EVP |
| *Portulaca sardoa* Danin, Bagella *et* Marrosu | Portulacaceae | exclusive EVP |
| *Potentilla crassinervia* Viv. | Rosaceae | insular EVP |
| *Potentilla rupestris* L. ssp. *corsica* (Soleir. *ex* Lehm.) Rouy *et* Camus | Rosaceae | insular EVP |
| *Prospero corsicum* (Boullu) J.M. Tison | Asparagaceae | insular EVP |
| *Ptychotis sardoa* Pignatti *et* Metlesics | Apiaceae | exclusive EVP |
| *Pulicaria vulgaris* Gaertn. var. *sardoa* Fiori | Asteraceae | exclusive EVP |
| *Quercus ichnusae* Mossa, Bacch. *et* Brullo | Fagaceae | exclusive EVP |
| *Ranunculus cordiger* Viv. ssp*. cordiger* | Ranunculaceae | insular EVP |
| *Ranunculus cordiger* Viv. ssp. *diffusus* (Moris) Arrigoni | Ranunculaceae | insular EVP |
| *Ranunculus cymbalarifolius* Balbis *et* Moris | Ranunculaceae | exclusive EVP |
| *Rhamnus persicifolia* Moris | Rhamnaceae | exclusive EVP |
| *Ribes multiflorum* Kit. *ex* Roem. *et* Schult. ssp. *sandalioticum* Arrigoni | Grossulariaceae | exclusive EVP |
| *Ribes sardoum* Martelli | Grossulariaceae | exclusive EVP |
| *Romulea bocchierii* Frignani *et* Iiriti | Iridaceae | exclusive EVP |
| *Romulea limbarae* Bég. *pro hybr.* | Iridaceae | exclusive EVP |
| *Romulea requienii* Parl. | Iridaceae | exclusive EVP |
| *Romulea revelieri* Jord. *et* Fourr. | Iridaceae | insular EVP |
| *Rubus arrigonii* Camarda | Rosaceae | exclusive EVP |
| *Rubus limbarae* Camarda | Rosaceae | exclusive EVP |
| *Rumex pulcher* L. ssp. *suffocatus* (Moris *ex* Bertol.) Nyman | Polygonaceae | exclusive EVP |
| *Rumex suffocatus* Moris *ex* Bertol. | Polygonaceae | exclusive EVP |
| *Ruta lamarmorae* Bacch., Brullo *et* Giusso del Galdo | Rutaceae | exclusive EVP |
| *Sagina pilifera* (DC.) Fenzl. | Caryophyllaceae | insular EVP |
| *Sagina revelieri* Jordan *et* Fourr. | Caryophyllaceae | insular EVP |
| *Salix arrigonii* Brullo | Salicaceae | exclusive EVP |
| *Salvia desoleana* Atzei *et* Picci | Lamiaceae | exclusive EVP |
| *Santolina corsica* Jord. *et* Fourr. | Asteraceae | insular EVP |
| *Santolina insularis* (Gennari *ex* Fiori) Arrigoni | Asteraceae | exclusive EVP |
| *Saponaria alsinoides* (Viv.) Viv. | Caryophyllaceae | insular EVP |
| *Saxifraga cervicornis* Viv. | Saxifragaceae | insular EVP |
| *Saxifraga corsica* (Ser. *ex* Duby) Gren*. et* Godr. | Saxifragaceae | insular EVP |
| *Scorzonera callosa* Moris | Asteraceae | exclusive EVP |
| *Scrophularia morisii* Vals. | Scrophulariaceae | exclusive EVP |
| *Scrophularia oblongifolia* Loisel. ssp. *oblongifolia* | Scrophulariaceae | insular EVP |
| *Scrophularia trifoliata* L. | Scrophulariaceae | insular EVP |
| *Sedum villosum* L. ssp. *glandulosum* (Moris) P. Fourn. | Crassulaceae | exclusive EVP |
| *Senecio siculus* All. var. *nemoralis* (Gennari) Pignatti | Asteraceae | exclusive EVP |
| *Senecio squalidus* ssp. *sardous* (Fiori) Greuter | Asteraceae | exclusive EVP |
| *Senecio transiens* (Rouy) Jeanm. | Asteraceae | insular EVP |
| *Senecio vulgaris* L. var. *tyrrhenus* Fiori | Asteraceae | exclusive EVP |
| *Seseli praecox* (Gamisans) Gamisans | Apiaceae | insular EVP |
| *Sesleria insularis* Sommier ssp. *barbaricina* Arrigoni | Poaceae | exclusive EVP |
| *Sesleria insularis* Sommier ssp. *morisiana* Arrigoni | Poaceae | exclusive EVP |
| *Silene beguinotii* Vals. | Caryophyllaceae | exclusive EVP |
| *Silene ichnusae* Brullo, De Marco *et* De Marco *fil.* | Caryophyllaceae | exclusive EVP |
| *Silene martinolii* Bocchieri *et* Mulas | Caryophyllaceae | exclusive EVP |
| *Silene morisiana* Bég. *et* Rav. | Caryophyllaceae | exclusive EVP |
| *Silene nodulosa* Viv. | Caryophyllaceae | insular EVP |
| *Silene requienii* Otth | Caryophyllaceae | insular EVP |
| *Silene rosulata* Soy-Will. *et* Godr. ssp. *sanctae-theresiae* (Jeanm.) Jeanm. | Caryophyllaceae | exclusive EVP |
| *Silene succulenta* Forssk. ssp. *corsica* (DC.) Nyman | Caryophyllaceae | insular EVP |
| *Silene valsecchiae* Bocchieri | Caryophyllaceae | exclusive EVP |
| *Silene velutina* Pourr. *et* Loisel. | Caryophyllaceae | insular EVP |
| *Solenopsis minuta* (L.) C. Presl ssp. *corsica* Meikle | Campanulaceae | insular EVP |
| *Stachys corsica* Pers. | Lamiaceae | insular EVP |
| *Stachys corsica* Pers. var. *micrantha* Bertol. | Lamiaceae | insular EVP |
| *Stachys glutinosa* L. | Lamiaceae | insular EVP |
| *Stachys salisii* Jord. *et* Fourr. | Lamiaceae | insular EVP |
| *Tanacetum audibertii* (Req.) DC. | Asteraceae | insular EVP |
| *Taraxacum genargenteum* Arrigoni | Asteraceae | exclusive EVP |
| *Thesium italicum* A. DC. *in* DC. | Santalaceae | exclusive EVP |
| *Thlapsi brevistylum* Jord. | Brassicaceae | insular EVP |
| *Thymus herba-barona* Loisel. ssp. *herba-barona* | Lamiaceae | insular EVP |
| *Trisetaria gracile* (Moris) Banfi *et* Arrigoni | Poaceae | insular EVP |
| *Urtica atrovirens* Req. *ex* Loisel. ssp. *atrovirens* | Urticaceae | insular EVP |
| *Verbascum conocarpum* Moris ssp. *conocarpum* | Scrophulariaceae | insular EVP |
| *Verbascum plantagineum* Moris | Scrophulariaceae | exclusive EVP |
| *Veronica verna* L. ssp. *brevistyla* (Moris) Rouy | Plantaginaceae | insular EVP |
| *Vinca sardoa* (Stearn) Pignatti | Apocynaceae | exclusive EVP |
| *Viola corsica* Nym. ssp. *limbarae* Merxm. *et* Lippert | Violaceae | exclusive EVP |
