# Supplementary material for: Disentangling the influence of environmental and anthropogenic factors on the distribution of endemic vascular plants in Sardinia
Source: PLoS One. 2017 Aug 2;12(8):e0182539. doi: 10.1371/journal.pone.0182539 (PMC5540478; doi:10.1371/journal.pone.0182539)
Supplement: S2 Table — (DOCX) [file pone.0182539.s002.docx]

**S2 Table. Bivariate correlation matrix of Endemic Vascular Plant Species Richness (EVPR) with all explanatory variables and of explanatory variables with each other.**

Complete bivariate correlation matrix of total (Tot), spread and exclusive (Excl.) Endemic Vascular Plant Species Richness (EVPR), with explanatory variables and of explanatory variables with each other. Values are given as the correlation coefficient r resulting from polyserial test. Values in bold were not excluded from further calculations. Class assigns each variable to three general categories: Human influence (H), Topography (T) and Climate (C).

|  |  | Tot | Insular | Excl | HII | Fires | LU_ratio | Build | Roads | N_Geol | N_Land | Elev | Slope | Lit_type | Bio1 | Bio6 | Bio7 | Bio12 | Bio15 | Bio17 |
| --- | --- | --- | --- | --- | --- | --- | --- | --- | --- | --- | --- | --- | --- | --- | --- | --- | --- | --- | --- | --- |
| HII | H | **-0,22** | **-0,24** | **-0,11** | 1,00 |  |  |  |  |  |  |  |  |  |  |  |  |  |  |  |
| Fires | H | -0,09 | **-0,10** | -0,05 | 0,28 | 1,00 |  |  |  |  |  |  |  |  |  |  |  |  |  |  |
| LU_ratio | H | **-0,29** | **-0,31** | **-0,16** | 0,53 | 0,33 | 1,00 |  |  |  |  |  |  |  |  |  |  |  |  |  |
| Build | H | -0,07 | -0,08 | -0,02 | 0,22 | 0,10 | 0,18 |  |  |  |  |  |  |  |  |  |  |  |  |  |
| Roads | H | **-0,11** | **-0,13** | -0,04 | 0,30 | 0,29 | 0,39 | 0,28 | 1,00 |  |  |  |  |  |  |  |  |  |  |  |
| N_Geol | E | -0,12 | **-0,13** | -0,08 | 0,09 | 0,18 | 0,22 | 0,10 | 0,37 | 1,00 |  |  |  |  |  |  |  |  |  |  |
| N_Land | E | **-0,16** | **-0,18** | -0,08 | 0,24 | 0,08 | 0,30 | 0,01 | 0,08 | -0,02 | 1,00 |  |  |  |  |  |  |  |  |  |
| Elev | E | **0,54** | **0,58** | **0,30** | -0,36 | -0,18 | -0,50 | -0,12 | -0,21 | -0,14 | -0,24 | 1,00 |  |  |  |  |  |  |  |  |
| Slope | E | **0,24** | **0,27** | **0,10** | -0,47 | -0,26 | -0,63 | -0,14 | -0,28 | -0,03 | -0,31 | 0,39 | 1,00 |  |  |  |  |  |  |  |
| Lit_Type | E | -0,09 | -0,09 | -0,05 | 0,12 | 0,04 | 0,16 | -0,01 | 0,02 | 0,05 | 0,22 | -0,12 | -0,28 | 1,00 |  |  |  |  |  |  |
| Bio1 | C | -0,53 | -0,57 | -0,30 | 0,34 | 0,18 | 0,48 | 0,11 | 0,20 | 0,13 | 0,22 | **-0,99** | -0,35 | 0,11 | 1,00 |  |  |  |  |  |
| Bio6 | C | -0,50 | -0,54 | -0,28 | 0,27 | 0,13 | 0,39 | 0,09 | 0,17 | 0,14 | 0,13 | **-0,95** | -0,27 | 0,08 | **0,97** | 1,00 |  |  |  |  |
| Bio7 | C | **0,14** | **0,17** | 0,04 | 0,06 | 0,11 | 0,06 | -0,02 | -0,03 | -0,09 | 0,25 | 0,41 | -0,07 | 0,07 | -0,38 | -0,58 | 1,00 |  |  |  |
| Bio12 | C | 0,30 | 0,33 | 0,17 | -0,19 | -0,16 | -0,26 | -0,09 | -0,13 | -0,06 | 0,13 | **0,75** | 0,11 | 0,14 | **-0,79** | **-0,78** | 0,38 | 1,00 |  |  |
| Bio15 | C | **-0,27** | **-0,30** | **-0,11** | 0,29 | 0,09 | 0,40 | 0,04 | 0,13 | 0,03 | 0,60 | -0,50 | -0,42 | 0,36 | 0,49 | 0,45 | -0,04 | 0,04 | 1,00 |  |
| Bio17 | C | 0,39 | 0,43 | 0,20 | -0,30 | -0,19 | -0,41 | -0,10 | -0,17 | -0,03 | -0,23 | **0,89** | 0,29 | -0,04 | **-0,92** | **-0,87** | 0,26 | **0,82** | -0,47 | 1,00 |

Abbreviations of explanatory variables: HII: Human Influence Index; Fires: index of fires occurred among the years 2005-2013; LU_ratio: 1-2 Land Use first levels (i.e. anthropic uses) and the total surface; Build: number of buildings; Roads: kilometres of roads per grid; Elev: Elevation; Slope: slope; Lit_Type: lithological types; Bio1: annual mean temperature; Bio6: minimum temperature of the coldest month; Bio7: annual range of temperature; Bio12: annual precipitation; Bio15: precipitation seasonality; Bio17: precipitation of driest quarter.
