# Supplementary material for: Disentangling the influence of environmental and anthropogenic factors on the distribution of endemic vascular plants in Sardinia
Source: PLoS One. 2017 Aug 2;12(8):e0182539. doi: 10.1371/journal.pone.0182539 (PMC5540478; doi:10.1371/journal.pone.0182539)
Supplement: S3 Table — (DOCX) [file pone.0182539.s003.docx]

**S3 Table.**  **Fractions of variation explained by climate, topography and human factors**

**S3 Table** Details of fractions explained by Climate [C], Topography [T] and Human [H] factor subsets. Coefficients of fractions explained are the deviance values (D^2^) [1] of GLM with one [C], [T], [H], two [C + T], [T + H] and all [C + T + H] groups of explanatory variables. The D^2^ is the deviance accounted for by each model and it is considered the equivalent of the coefficient of determination (R^2^) of linear regressions [2]*.*These values were computed using the function *Dsquared* of the R package modEvA [1]. Procedures for computation of these measures were done following the manual of the same modEvA package [2] and references therein. Results were used to perform the *varPart* function of the R package modEvA [2].

|  | **[C + T]** | **[T + H]** | **[C + H]** | **[C]** | **[T]** | **[H]** | **[U]** | **Total explained deviance [C + T + H]** |
| --- | --- | --- | --- | --- | --- | --- | --- | --- |
| **Total EVPR** | 0,286 | 0,281 | 0.126 | 0,081 | 0,278 | 0.084 | 0,711 | 0,289 |
| **Insular EVPR** | 0,323 | 0,319 | 0.153 | 0,102 | 0,316 | 0.099 | 0,674 | 0,326 |
| **Exclusive EVPR** | 0,115 | 0,113 | 0.062 | 0,045 | 0,111 | 0.032 | 0,883 | 0,117 |

**References**

1. Guisan A., Zimmermann NE. Predictive habitat distribution models in ecology. Ecol Model. 2000; 135: 147–186.

2. Barbosa AM, Brown JA, Jiménez-Valverde A, Real R. modEvA: Model Evaluation and Analysis. R package, version 1.3.3; 2017.
