# Supplementary material for: Disentangling the influence of environmental and anthropogenic factors on the distribution of endemic vascular plants in Sardinia
Source: PLoS One. 2017 Aug 2;12(8):e0182539. doi: 10.1371/journal.pone.0182539 (PMC5540478; doi:10.1371/journal.pone.0182539)
Supplement: S4 Table — (DOCX) [file pone.0182539.s004.docx]

**S4 Table. Total and proportion of variance explained by each explanatory variable.**

**S4 Table** Total of variance explained by GLM full models and proportion of variance explained by each variable belonging to Climate (C), Topography (T) or Human (H) group; normalized percentages (%) are reported for each variable used. Only statistically significant variable was considered, according to results shown in Table 1. Sums per each group are also reported for total, spread and exclusive EVPR. Results correspond to Figure 1a-f, in the main text.

|  | H (Excl EVPR = 0.02; insular EVPR = 0.041; tot EVPR = 0.03) | | | | T (Excl EVPR = 0.08; insular EVPR = 0.24; tot EVPR = 0.20) | | | | C (Excl EVPR = 0.01; insular EVPR = 0.04; tot EVPR = 0.03) | | |
| --- | --- | --- | --- | --- | --- | --- | --- | --- | --- | --- | --- |
|  | **HII** | **Fires** | **LU_ratio** | **Roads** | **N_Geol** | **N_Land** | **Elev** | **Slope** | **Bio7** | **Bio15** | **Total deviance explained** |
| **Exclusive EVPR** | - | - | 0.010(8.9%) | - | - | - | 0.071 (62.2%) | 0.007(6.0%) | - | 0.010(15.4%) | 0.117 |
| **Insular EVPR** | 0.012 (3.8%) | 0.002 (0.6%) | 0.025 (7.7%) | 0.002 (0.7%) | - | 0.007 (2.2%) | 0.216 (66.0%) | 0.013 (4.1%) | 0.016 (4.9%) | 0.023 (7.2%) | 0.327 |
| **Total EVPR** | 0.011 (3.69%) | - | 0.022 (7.7%) | 0.002 (0.7%) | - | 0.007 (2.3%) | 0.196 (67.0%) | 0.010 (3.6%) | 0.014 (4.7%) | 0.019 (6.69%) | 0.289 |

Abbreviations of explanatory variables: HII: Human Influence Index; Fires: index of fires occurred among the years 2005-2013; LU_ratio: 1-2 Land Use first levels (i.e. anthropic uses) and the total surface; Roads: kilometres of roads per grid; Elev: Elevation; Slope: slope; Bio7: annual range of temperature; Bio15: precipitation seasonality.
